# Early Recurrent Ischemic Lesions in Patients With Cryptogenic Stroke and Patent Foramen Ovale: An Observational Study

**DOI:** 10.3389/fneur.2018.00996

**Published:** 2018-11-22

**Authors:** Tim Bastian Braemswig, Tatiana Usnich, Jan F. Scheitz, Hebun Erdur, Jochen B. Fiebach, Heinrich J. Audebert, Matthias Endres, Christian H. Nolte

**Affiliations:** ^1^Klinik und Hochschulambulanz für Neurologie, Charité–Universitätsmedizin Berlin, Corporate Member of Freie Universität Berlin, Humboldt-Universität zu Berlin, Berlin Institute of Health, Berlin, Germany; ^2^Berlin Institute of Health (BIH), Berlin, Germany; ^3^Center for Stroke Research Berlin (CSB), Charité – Universitätsmedizin Berlin, Berlin, Germany; ^4^German Centre for Cardiovascular Research (DZHK), Partner Site, Berlin, Germany; ^5^German Center for Neurodegenerative Diseases (DZNE), Partner Site, Berlin, Germany

**Keywords:** patent foramen ovale, stroke, MRI–magnetic resonance imaging, diffusion-weighted (DW) imaging, new ischemic lesions

## Abstract

**Background:** Randomized controlled trials indicate that patent foramen ovale (PFO) closure reduces risk of stroke recurrence in patients with cryptogenic stroke and PFO. However, the optimal time point for PFO closure is unknown and depends on the risk of stroke recurrence.

**Objective:** We aimed to investigate risk of early new ischemic lesions on cerebral magnetic resonance imaging (MRI) in cryptogenic stroke patients with and without PFO.

**Methods:** Cryptogenic stroke patients underwent serial MRI examinations within 1 week after symptom onset to detect early new ischemic lesions. Diffusion-weighted imaging (DWI) lesions were delineated, co-registered, and analyzed visually for new hyperintensities by raters blinded to clinical details. A PFO was classified as stroke-related in patients with PFO and a Risk of Paradoxical Embolism (RoPE) score >5 points.

**Results:** Out of 80 cryptogenic stroke patients, risk of early recurrent DWI lesions was not significantly different in cryptogenic stroke patients with and without PFO. Similar results were observed in patients ≤60 years of age. Patients with a stroke-related PFO even had a significantly lower risk of early recurrent ischemic lesions compared to all other patients with cryptogenic stroke (unadjusted odds ratio 0.23 [95% confidence interval 0.06–0.87], *P* = 0.030).

**Conclusion:** Our data argue against a high risk of early stroke recurrence in patients with cryptogenic stroke and PFO.

## Introduction

In the general population, prevalence of patent foramen ovale (PFO) is ~25% (1). Prevalence of PFO in patients with cryptogenic stroke is significantly higher than in the general population (2–5). In these patients, PFO is considered a possible etiology of stroke. The suggested pathophysiologic mechanisms include paradoxical embolism and local intraseptal thrombosis (5–9).

Randomized controlled trials indicate that patent foramen ovale (PFO) closure combined with antiplatelet therapy compared to antiplatelet therapy alone significantly reduces risk of stroke recurrence in young patients with cryptogenic stroke and PFO (10–12). Evidence in favor of PFO closure raises the question whether PFO closure is an urgent matter (13). However, the optimal time point for PFO closure is unknown and depends on the risk of stroke recurrence (14). New diffusion-weighted imaging (DWI) lesions after acute ischemic stroke are a sensitive marker for new ischemic events and are detected more frequently than clinical stroke recurrence alone (15–20). Here we analyzed, whether presence of PFO in cryptogenic stroke patients is associated with occurrence of early recurrent DWI lesions within 1 week after stroke.

## Materials and methods

### Study design and patients

We performed a *post-hoc* analysis of data drawn from an observational study conducted by the Center for Stroke Research Berlin (CSB) at the Charité—Universitätsmedizin Berlin, Campus Benjamin Franklin (Berlin, Germany; clinicaltrials.gov: NCT00715533). The study included acute ischemic stroke patients that were able to undergo MRI within 24 h after symptom onset (16–18, 21). We included patients recruited between March, 2008 and December, 2010 with a complete set of three MRI examinations within the first week after symptom onset and an undetermined etiology of stroke according to Trial of Org 10172 in Acute Stroke Treatment (TOAST) criteria (22). Diagnostic work-up in all patients included MRI, MR-angiography, carotid ultrasonography and cardiac rhythm monitoring for at least 24 h. Patients with multiple potential causes of stroke (22) and patients who underwent endovascular revascularization procedures were excluded. We excluded patients who underwent endovascular revascularization procedures, because endovascular procedures may cause new DWI lesions on MRI (23, 24). Patients who received thrombolysis were not excluded. All patients included in this study received standard stroke unit care following the guidelines of the European Stroke Organization (ESO) and the German Stroke Society (DSG; https://www.dsg-info.de/stroke-units/stroke-units-uebersicht.html). None of the patients included in this study underwent PFO closure during the study period. The study was approved by the local Ethics Committee (EA4/026/08). All patients gave written informed consent.

### MRI

Details have been reported previously (18, 21). In short, we conducted three cerebral MRI examinations on a 3-Tesla MRI scanner (Tim Trio, Siemens Medical, Erlangen, Germany): on admission, on the following day, and 4 to 7 days after symptom onset. DWI images were pseudonymized and afterwards reviewed in random order. Raters were blinded to clinical information. Hyperintensities on initial DWIs were delineated manually and then co-registered. Co-registered DWIs were analyzed visually for new hyperintensities through slice-by-slice comparison of the first and second, as well as the second and third DWI. New hyperintensities had to be clearly separate from the index lesion. All new diffusion hyperintensities regardless of apparent diffusion coefficient value were considered (17, 18, 21) Number of new DWI lesions was counted.

### Clinical data

Sociodemographic and laboratory data were collected from the medical records. All patients were assessed for stroke severity directly before the first MRI examination by physicians certified to assess the National Institutes of Health Stroke Scale (NIHSS) (25). PFO was diagnosed by transesophageal echocardiography (TEE) or transcranial Doppler. Both techniques have similar sensitivity and specificity (5, 26, 27). An associated atrial septum aneurysm (ASA) was diagnosed in patients with septum primum excursion >10 mm on TEE (11). The Risk of Paradoxical Embolism (RoPE) score was used to differentiate between patients with a high probability of a stroke-related PFO (high attributable risk) vs. an incidental PFO.(26, 28) The RoPE score is externally validated (28, 29) and varies from 0 to 9 points with higher scores indicating a higher attributable risk. In stroke patients with PFO and a RoPE score >5 points, the PFO has an attributable risk for stroke of 62% or more (28). Therefore in this study, patients with an undetermined etiology of stroke (22), PFO and a RoPE score >5 points were assumed to have a stroke-related PFO.

### Statistics

For comparisons of nominal and categorical variables, we used unadjusted, univariate logistic regression to obtain odds ratios (OR) and corresponding 95% confidence intervals (CI). Additionally, we used chi-square test to compare (1) patients with and without data available regarding PFO and (2) patients with and without tested PFO parameters (any PFO, stroke-related PFO, PFO in patients ≤60 years of age) regarding appearance of new DWI lesions. For comparisons of continuous variables, we used the Mann–Whitney *U*-test. Statistical significance was determined at an alpha level of 0.05 (16–18, 21).

## Results

**Figure 1:**
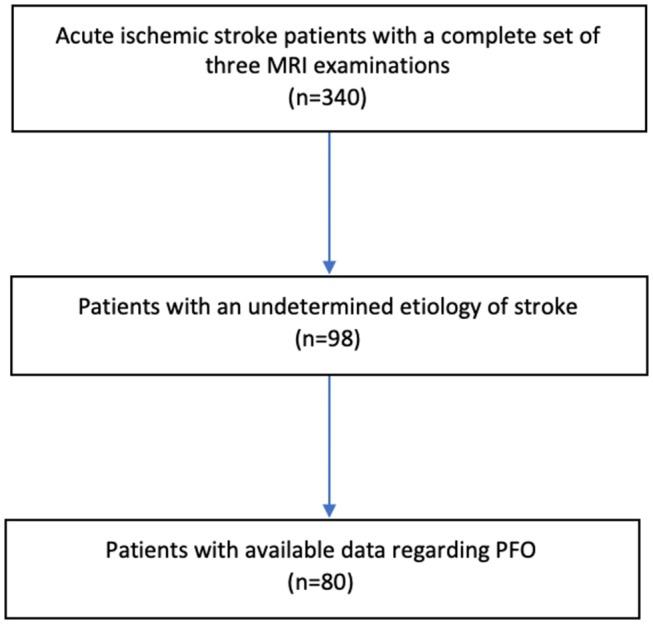
Data flow diagram.

Out of 340 acute ischemic stroke patients examined, 98 patients (29%) had a cryptogenic stroke defined as stroke of undetermined etiology. Of these, 80 patients had data available regarding PFO and constitute the study population (Figure 1, Table 1). Cryptogenic stroke patients with and without data available regarding PFO did not differ regarding sex and NIHSS > 3 points. Patients without data available regarding PFO were more often > 60 years of age (88.9 vs. 57.5%, *p* = 0.013).

**Table 1:** Sociodemographic and clinical characteristics of the study population.

**Characteristic**	
Sex (female), n (%)	29 (36%)
Age (years), median (IQR)	65 (49–72)
NIHSS, median (IQR)	2.5 (1–5)
Diabetes mellitus, n (%)	11 (14%)
Arterial hypertension, n (%)	46 (58%)
CHD, n (%)	5 (6%)
Previous stroke, n (%)	16 (20%)
Prior antiplatelet therapy, n (%)	16 (20%)
Thrombolysis, n (%)	18 (23%)
Antiplatelet therapy during hospital stay, n (%) ^a^	73 (91%)
Anticoagulation during hospital stay, n (%) ^b^	4 (5%)
Any PFO, n (%)	32 (40%)
PFO + ASA, n (%) ^c^	11 (14%)
PFO + RoPE score > 5 points, n (%)	18 (23%)

NIHSS indicates National Institutes of Health Stroke Scale; CHD, coronary heart disease; PFO, patent foramen ovale; and ASA, atrial septal aneurysm.

a The variable antiplatelet therapy during hospital stay was known in 77/80 patients.

b The variable anticoagulation during hospital stay was known in 77/80 patients.

c *The variable PFO + ASA was known in 77/80 patients*.

Thirty-two out of 80 patients (40%) had any PFO and 18/80 patients (23%) with PFO had a RoPE score >5 points (stroke-related PFO). Early recurrent DWI lesions were detected in 11 of 32 patients (34%) with any PFO and in 3 of 18 patients (17%) with a stroke-related PFO.

Neither any PFO (unadjusted OR 0.67 [95%CI 0.27–1.70], *p* = 0.403; 34% [with PFO] vs. 44% [without PFO], *p* = 0.402) nor stroke-related PFO were significantly positively associated with early recurrent DWI lesions. On the contrary, stroke-related PFO was negatively associated with early recurrent DWI lesions (unadjusted OR 0.23 [95%CI 0.06–0.87], *p* = 0.030; 17% [with stroke-related PFO] vs. 47% [without PFO or with incidental PFO], *p* = 0.022). Factors associated with early recurrent DWI lesions were diabetes mellitus, arterial hypertension, and older age (Table 2).

**Table 2:** Sociodemographic and clinical characteristics of the study population by the presence vs. absence of early recurrent DWI lesions.

**Characteristic**	**Unadjusted OR** **(95% CI)**	***p***
Sex (female)	0.87 (0.34 – 2.22)	0.776
Age > 60 years	**4.59 (1.67** – **12.65)**	**0.003**
NIHSS > 3 points	0.96 (0.39 – 2.38)	0.926
Diabetes mellitus	**5.00 (1.21** – **20.61)**	**0.026**
Arterial hypertension	**3.55 (1.33** – **9.46)**	**0.011**
CHD	1.00 (0.16 – 6.35)	1.000
Previous stroke	2.29 (0.75 – 6.97)	0.144
Thrombolysis	1.70 (0.59 – 4.88)	0.328
Glucose > 10 mmol/l ^a^	2.35 (0.49 – 11.34)	0.289
LDL > 3.4 mmol/l ^b^	1.17 (0.45 – 3.03)	0.752
Antiplatelet therapy during hospital stay ^c^	0.70 (0.09 – 5.23)	0.726
Anticoagulation during hospital stay ^d^	1.43 (0.19 – 10.75)	0.726
Any PFO	0.67 (0.27 – 1.70)	0.403
PFO + RoPE score > 5 points	**0.23 (0.06** – **0.87)**	**0.030**
Any PFO in patients ≤60 years of age ^e^	0.44 (0.08 – 2.39)	0.342

NIHSS indicates National Institutes of Health Stroke Scale; CHD, coronary heart disease; LDL, low-density lipoprotein; PFO, patent foramen ovale; and RoPE, Risk of Paradoxical Embolism score.

a The variable glucose was known in 76/80 patients.

b The variable LDL was known in 74/80 patients.

c The variable antiplatelet therapy during hospital stay was known in 77/80 patients.

d The variable anticoagulation during hospital stay was known in 77/80 patients.

e *The variable any PFO in patients ≤60 years of age was known in 34 patients. Bold values are statistically significant results*.

Out of the 80 patients with cryptogenic stroke, 34 patients were ≤60 years of age (29% women; median age [years]: 48 [interquartile range {IQR}, 41–52]; median NIHSS: 2 [IQR, 1–4]). Of these, 20 patients (59%) had a PFO (18/20 patients had a stroke-related PFO). Early recurrent DWI lesions were detected in 3 of 20 patients (15%) with PFO. PFO was not positively associated with early recurrent DWI lesions [unadjusted OR 0.44 [95%CI 0.08–2.39], *p* = 0.342; 15% [with PFO] vs. 29% [without PFO], *p* = 0.335].

Results did not change when PFO and an associated atrial septal aneurysm (ASA) was taken into account (PFO+ASA: unadjusted OR 0.51 [95%CI 0.12–2.09], *p* = 0.349; PFO+ASA in patients ≤60 years of age: unadjusted OR 0.54 [95%CI 0.05 – 5.50], *p* = 0.605).

The number of early recurrent DWI lesions was significantly lower in cryptogenic stroke patients with any PFO (32/80) compared to patients without PFO (median: 2 [IQR, 1-2] vs. 5 [IQR, 2–8]; *p* = 0.014).

## Discussion

In this population of patients with cryptogenic stroke undergoing serial MRI examinations, PFO was not associated with early recurrent ischemic lesions.

Risk of stroke recurrence may vary over time. For example, in stroke patients with symptomatic carotid stenosis, endarterectomy should be done within the first 2 weeks after the initial event because the survival curve for recurrence is front-loaded (30, 31).

In general stroke cohorts, early recurrent ischemic lesions are reported to appear in 24–34% of patients while higher rates are reported, for example, in acute stroke patients with large artery atherosclerosis (20). In this study, frequency of early recurrent ischemic lesions was lower both in patients with a stroke-related PFO (17%) and in young stroke patients ≤60 years of age with PFO (15%). In all tested conditions (any PFO, stroke-related PFO, PFO in patients ≤60 years of age) presence of PFO was not positively associated with early recurrent ischemic lesions. In the CLOSE trial (Patent Foramen Ovale Closure or Anticoagulants vs. Antiplatelet Therapy to Prevent Stroke Recurrence) (11) only stroke patients with PFO and additional echocardiographic features like ASA were included. Therefore, we included PFO and an associated ASA as an additional parameter but lack of association regarding early recurrent ischemic lesions remained. Patients with a stroke-related PFO even had a significantly lower risk of early recurrent ischemic lesions compared to all other patients with cryptogenic stroke (patients without PFO and patients with an incidental PFO)–this result complements a previous study that reported a low long-term risk of a clinically diagnosed stroke recurrence in patients with a stroke-related PFO (28). In addition, number of early recurrent ischemic lesions in patients with any PFO was significantly lower compared to patients without PFO. In contrast to PFO, well-known risk factors for both clinically overt and silent stroke recurrence like diabetes mellitus, arterial hypertension, and older age (32, 33) were associated with an increased risk of early recurrent ischemic lesions in this cohort.

Limitations of this study have to be considered. First, this is a single-center *post-hoc* analysis. Second, the number of patients was small. We cannot exclude a type-2 error with respect to non-significant findings. Still, the point estimates argue against a clinically relevant, increased risk of early recurrent ischemic lesions in patients with PFO. Patients with a stroke-related PFO had a significantly lower risk of early recurrent ischemic lesions. In addition, the number of early recurrent ischemic lesions in patients with any PFO was significantly lower.

In conclusion, our data argue against a high risk of early stroke recurrence in patients with cryptogenic stroke and PFO, especially in patients eligible for PFO closure (≤60 years of age, patients with PFO and an associated ASA). Therefore, our findings suggest that PFO closure does not necessarily have to be performed early after the initial stroke. Rather a comprehensive clinical assessment to exclude other potential causes of stroke should be prioritized.

## Data availability statement

Anonymized data will be shared by request from any qualified investigator for analysis at the Department of Neurology, Charité–Universitätsmedizin Berlin, Berlin. Data sharing will be restricted to non-commercial and academic purposes only. The corresponding author will keep a copy of the final data set for at least 10 years.

## Author contributions

TBB: conception/design of the work, acquisition of data, data analysis and interpretation, drafting the article, and final approval; TU: acquisition of data, data interpretation, critical review, and final approval; JFS: data analysis and interpretation, critical review, and final approval; HE: data analysis and interpretation, critical review, and final approval; JBF: acquisition of data, data interpretation, critical review, and final approval; HJA: data interpretation, critical review, and final approval; ME: data interpretation, critical review, and final approval; CHN: conception/design of the work, data analysis and interpretation, critical review, and final approval.

### Conflict of interest statement

TBB has received travel support from W. L. Gore and Associates. JFS has received a speaker's honorarium from W. L. Gore and Associates. CHN has received speaker's honoraria from W. L. Gore and Associates. The remaining authors declare that the research was conducted in the absence of any commercial or financial relationships that could be construed as a potential conflict of interest.
